# Etiology of Pediatric Meningitis in West Africa Using Molecular Methods in the Era of Conjugate Vaccines against Pneumococcus, Meningococcus, and *Haemophilus influenzae* Type b

**DOI:** 10.4269/ajtmh.19-0566

**Published:** 2020-05-26

**Authors:** Brenda A. Kwambana-Adams, Jie Liu, Catherine Okoi, Jason M. Mwenda, Nuredin I. Mohammed, Enyonam Tsolenyanu, Lorna Awo Renner, Daniel Ansong, Beckie N. Tagbo, Muhammad F. Bashir, Mamadou Kourna Hama, Mouhamadou A. Sonko, Jean Gratz, Archibald Worwui, Peter Ndow, Adam L. Cohen, Fatima Serhan, Richard Mihigo, Martin Antonio, Eric Houpt, on behalf of the Paediatric Bacterial Meningitis Surveillance Network in West Africa

**Affiliations:** 1 WHO Collaborating Centre for New Vaccines Surveillance, Medical Research Council Unit The Gambia at London School of Hygiene and Tropical Medicine, Banjul, The Gambia;; 2 Division of Infection and Immunity, NIHR Global Health Research Unit on Mucosal Pathogens, University College London, London, United Kingdom;; 3 Division of Infectious Diseases and International Health, Department of Medicine, University of Virginia, Charlottesville, Virginia;; 4 World Health Organization (WHO), Regional Office for Africa, Brazzaville, Congo;; 5 Department of Paediatrics, Sylvanus Olympio Teaching Hospital, Lomé, Togo;; 6 University of Ghana School of Medicine and Dentistry, Accra, Ghana;; 7 Komfo Anokye Teaching Hospital, Kumasi, Ghana;; 8 Institute of Child Health, University of Nigeria Teaching Hospital, Enug, Nigeria;; 9 Department of Paediatrics, University of Nigeria Teaching Hospital Ituku-Ozalla, Enug, Nigeria;; 10 Department of Paediatrics, Abubakar Tafawa Balewa University Teaching Hospital, Bauchi, Nigeria;; 11 Laboratoire, Hopital National de Niamey, Niamey, Niger;; 12 Centre Hospitalier National d’Enfants Albert Royer, Dakar, Senegal;; 13 World Health Organization, Geneva, Switzerland;; 14 Division of Microbiology and Immunity, Warwick Medical School, University of Warwick, Coventry, United Kingdom;; 15 Department of Infection Biology, Faculty of Infectious and Tropical Diseases, London School of Hygiene and Tropical Medicine, London, United Kingdom

## Abstract

Despite the implementation of effective conjugate vaccines against the three main bacterial pathogens that cause meningitis, *Streptococcus pneumoniae*, *Haemophilus influenzae* type b (Hib), and *Neisseria meningitidis* serogroup A, the burden of meningitis in West Africa remains high. The relative importance of other bacterial, viral, and parasitic pathogens in central nervous system infections is poorly characterized. Cerebrospinal fluid (CSF) specimens were collected from children younger than 5 years with suspected meningitis, presenting at pediatric teaching hospitals across West Africa in five countries including Senegal, Ghana, Togo, Nigeria, and Niger. Cerebrospinal fluid specimens were initially tested using bacteriologic culture and a triplex real-time polymerase chain reaction (PCR) assay for *N. meningitidis*, *S. pneumoniae*, and *H. influenzae* used in routine meningitis surveillance*.* A custom TaqMan Array Card (TAC) assay was later used to detect 35 pathogens including 15 bacteria, 17 viruses, one fungus, and two protozoans. Among 711 CSF specimens tested, the pathogen positivity rates were 2% and 20% by the triplex real-time PCR (three pathogens) and TAC (35 pathogens), respectively. TAC detected 10 bacterial pathogens, eight viral pathogens, and *Plasmodium*. Overall, *Escherichia coli* was the most prevalent (4.8%), followed by *S. pneumoniae* (3.5%) and *Plasmodium* (3.5%). Multiple pathogens were detected in 4.4% of the specimens. Children with human immunodeficiency virus (HIV) and *Plasmodium* detected in CSF had high mortality. Among 220 neonates, 17% had at least one pathogen detected, dominated by gram-negative bacteria. The meningitis TAC enhanced the detection of pathogens in children with meningitis and may be useful for case-based meningitis surveillance.

## INTRODUCTION

Meningitis is a life-threatening inflammatory disease of the brain and spinal cord that can be caused by bacterial, viral, fungal, and parasitic infections.^1^ The greatest burden occurs in sub-Saharan Africa, bearing half of the estimated 2.8 million cases and 58% of the 318,400 deaths estimated annually.^2^ Up to a quarter of bacterial meningitis survivors may be left with sequelae including cognitive impairment, paralysis, and hearing loss.^3^ Children younger than 5 years are disproportionately affected.^2^

Before the rollout of effective conjugate vaccines, *Neisseria meningitidis*, *Haemophilus influenzae* type B (Hib), and *Streptococcus pneumoniae* accounted for the bulk of meningitis cases in children.^2,4,5^ However, as vaccines for these three bacterial pathogens have been introduced across West Africa, the relative importance of other causes of meningitis such as gram-negative bacteria, ß-hemolytic streptococci, *Staphylococcus*, *Listeria*, fungi, and viruses has increased.^2^ The broad spectrum of meningeal pathogens, some of which require special therapy or should not receive antibiotics, poses new challenges for rapid and accurate diagnosis.

Historically, meningitis diagnosis and surveillance are hindered by the very low pathogen yields from bacteriologic culture. Mwenda and colleagues reported that culture confirmation was less than 1% among 48,284 children younger than 5 years with suspected meningitis across 26 African countries.^6^ Accordingly, the WHO has recommended improved diagnostics and surveillance as key priorities on their road map to eliminate epidemic meningitis in Africa by 2030.^7^ A technical task force identified the need for a multiplex diagnostic tool with the capacity to simultaneously detect meningeal pathogens. Real-time polymerase chain reaction (PCR)–based tools have the capacity to detect meningeal pathogens with high sensitivity, specificity, and reproducibility.^8–10^ Several meningitis/encephalitis molecular multiplex PCR assays have been developed, some of which are commercially available.

In this work, because of the ability to customize the pathogens we wished to detect, we used the TaqMan Array Card (TAC). TAC is Card is a compartmentalized probe-based real-time PCR system that uses microfluidic technology, with PCRs configured in a 384-well array format. TaqMan Array Card can be used to simultaneously and accurately detect bacterial, viral, protozoan, and fungal pathogens using very small volumes of the clinical specimen. TAC has been used to improve the diagnostic yield in other low-resource settings, for example, determining the etiology of febrile illness and sepsis in East Africa^11,12^ and South Africa.^13^ Saha and colleagues used the TAC platform to determine the etiology of community-acquired neonatal infections in South Asia.^14^ We have previously used the TAC platform to improve the case-level and population-level ascertainment of the causes of diarrhea in multiple countries.^15^

We therefore used a customized TAC assay that targets 35 meningitis and encephalitis pathogens for case ascertainment in cerebrospinal fluid (CSF) specimens collected from five West African countries among children younger than 5 years with suspected meningitis. Our study included suspected meningitis cases that occurred between 2017 and 2018, after the rollout of the pneumococcal conjugate vaccine (PCV), the meningococcal A protein–polysaccharide conjugate vaccine (MenAfriVac), and the Hib conjugate vaccine in all five countries. This study therefore allowed an examination of the meningitis pathogens that countries should prioritize.

## METHODS

### Surveillance design and sample collection.

The WHO Collaborating Center for New Vaccines Surveillance (WHO CC NVS) hosted at the Medical Research Council Unit, the Gambia, at the London School of Hygiene and Tropical Medicine (MRCG at LSHTM) also serves as the WHO Regional Reference laboratory (WHO RRL) for invasive bacterial diseases. The WHO CC and RRL support the WHO-coordinated Pediatric Bacterial Meningitis Surveillance (PBM) network across 10 countries in West and Central Africa, a region of the world with high under-five mortality. The WHO-coordinated PBM enrolled children younger than 5 years with suspected meningitis presenting at sentinel sites. The sentinel sites participating in the PBM network are pediatric teaching and referral hospitals. The six sentinel sites included in this study were in Senegal (Dakar),^16^ Ghana (Kumasi and Accra),^17^ Togo (Lome),^18^ Nigeria (Enugu),^19^ and Niger (Niamey),^20^ all countries at risk of meningitis epidemics.^21^ The sentinel sites in Niger and Senegal fall within the hyperendemic region of the meningitis belt. The sentinel sites were selected for the TAC study based on the quality of the surveillance data and the number of samples available for TAC testing.^6^

Suspected cases were any child aged 0–59 months admitted to a sentinel hospital conducting surveillance with a sudden onset of fever (> 38.5°C rectal or 38.0°C axillary) and one of the following signs: neck stiffness, altered consciousness (with no other alternative diagnosis), or other meningeal signs.^22,23^ Lumbar puncture was performed to obtain CSF from suspected cases. Standard bacteriology, cytology and biochemistry were performed on CSF specimens at the sentinel sites.^24^ The remaining CSF specimens were stored at −20°C until they were shipped on dry ice to the WHO RRL and stored at −80°C until testing. For this pilot, we tested all residual CSF with volumes of at least 150 µL collected between January 2017 and June 2018 from the sentinel sites.

### Ethics review and approvals.

The standard operating procedures and protocols for this pilot were approved by The joint Gambia Government/MRCG at LSHTM Ethics Committee. The TAC diagnostics were provided by the University of Virginia, VA, whose IRB approved the grant proposal. Approval was granted by the Ministries of Health of the participating countries to test CSF samples collected as part of ongoing routine surveillance for vaccine-preventable diseases.

### Laboratory procedures.

Triplex real-time PCR detection of *N. meningitidis*, *S. pneumoniae*, and *H. influenzae* was performed at the WHO CC NVS as described previously.^25,26^ For this pilot, we assessed etiologies using a custom-made TAC (Thermo Fisher Scientific, Carlsbad, CA) that compartmentalized probe-based quantitative polymerase chain reaction (qPCR) assays for 35 pathogens selected based on the global, regional, and national estimates of meningitis and sepsis.^2,27^ The TAC assay included 15 bacteria (*Bartonella* spp.; *Brucella* spp.; *Escherichia coli*/*Shigella* spp.; *H. influenzae*; Hib; *Klebsiella pneumoniae*; *Leptospira* spp.; *Listeria monocytogenes*; *Mycoplasma pneumoniae*; *Mycobacterium tuberculosis*; *N. meningitidis*; *N. meningitidis* serogroups A, B, C, W, X, and Y; *Rickettsia* spp.; *Salmonella enterica*; *Staphylococcus aureus*; *Streptococcus agalactiae*; and *S. pneumoniae*), 17 viruses (adenovirus, cytomegalovirus (CMV), dengue, Epstein–Barr virus (EBV), enterovirus, human herpesvirus 6, herpes simplex virus 1, herpes simplex virus 2, human immunodeficiency virus (HIV) I, HIV II, influenza A, measles, mumps, parechovirus, parvovirus B19, varicella zoster virus, and West Nile virus), one fungus (*Cryptococcus neoformans*), and two protozoans (*Plasmodium* spp., *Plasmodium falciparum*, and *Toxoplasma gondii*). Primer and probe sequences were adapted largely from Onyango and colleagues^28^ as well as from our prior work and other publications using the same platform.^11,29–31^ New assays developed and validated for this project are listed in Supplemental File 1. For each specimen, at least 150 µL but up to 200 µL of the CSF underwent nucleic acid extraction using QIAamp MinElute Virus Spin kit (Qiagen, Hilden, Germany) following the manufacturer’s instruction. Samples with less than 150 µL of the CSF were excluded. When the sample volume was less than 200 µL, 0.9% sodium chloride was supplemented to bring the volume to 200 µL. This affected < 3% of specimens tested. Two external controls, bacteriophage MS2 and phocine herpesvirus, were added to assess extraction and amplification efficiency. We included one extraction blank per batch of extraction to monitor contamination. Purified nucleic acids were subjected to TAC qPCR. The reaction mixes contained 25 µL of TaqMan Fast Virus 1-Step Master Mix and 75 µL of total nucleic acid extract or nuclease-free water as negative control. We included one negative control per 10 cards to exclude laboratory contamination. Amplification involved a reverse transcription step at 50°C for 10 minutes, and then an initial denaturation step at 95°C for 20 seconds, followed by 40 cycles of 95°C for 3 seconds and 60°C for 30 seconds. All detections with a quantification cycle (Cq) value greater than 35 were considered negative. Valid results required proper functioning of controls and excluded data flagged by the QuantStudio^™^ Real Time PCR software version 1.2(Thermo Fisher Scientific, Carlsbad, CA), and 99% of specimens had valid results. The WHO RRL participates in the WHO External Quality assessment program for the invasive bacterial vaccine preventable diseases, UK NEQAS for Microbiology (http://www.ukneqasmicro.org.uk).

### Statistical analysis.

Participant characteristics were summarized using proportions for categorical variables and medians for continuous variables. We examined associations using chi-square/Fisher’s exact and Mann–Whitney U test for categorical and continuous variables, respectively. Odds ratios were calculated to assess the pathogen association with mortality where possible. Accuracy was determined with reference to the triplex real-time PCR results. All tests were two-tailed, at 5% significance level. Analyses were performed using SPSS (version 25.0; IBM Corp, Armonk, NY) and Stata statistical software: Release 12 (StataCorp LP, College Station, TX).

## RESULTS

During the study period, a total of 1,535 suspected meningitis cases were reported across the sentinel sites, and 1,088 CSF specimens were sent to the WHO RRL for molecular testing. Reasons for CSF samples not being sent to the WHO RRL vary but include 1) lumbar puncture not performed, 2) insufficient volumes of CSF, and 3) missing CSF samples. Of the CSF samples received at the RRL, 717/1088 (65%) had sufficient volume to perform TAC analysis, and, of these, 711/717 (99%) yielded valid TAC results. The characteristics of patients with valid TAC results are shown in Table 1. The surveillance site in Ghana enrolled predominantly neonates (median age, < 1 month), whereas in the other sites, toddlers or young children accounted for the bulk of suspected cases (median ages at other sites, 11–36 months). A majority (67%) of children in Ghana reported having received antibiotics before lumbar puncture, whereas prior antibiotic treatment was less commonly reported at the other sites (0–35%). The CSF samples were collected on average 3 days after the onset of the illness. Most children did not have significant CSF pleocytosis (87% had CSF whole blood cell count (WBC) < 10). Among the 605 patients with outcome at discharge information, 9.8% died.

**Table 1:** Clinical characteristics of the suspected meningitis cases enrolled in surveillance

	Ghana	Nigeria	Niger	Senegal	Togo
Suspected meningitis cases reported*	351	177	320	146	541
CSF specimens tested by TAC†	205	139	31	31	305
Gender					
Male, *n* (%)	115 (56)	82 (59)	18 (58)	19 (61)	176 (58)
Age (months)‡					
Median (months)	0.6	23	36	11	16
< 1, *n* (%)	123 (60)	10 (7.2)	0	8 (26)	77 (25)
1–23, *n* (%)	59 (29)	55 (40)	11 (36)	13 (42)	100 (33)
24–59, *n* (%)	22 (11)	74 (53)	20 (65)	10 (32)	128 (42)
Collection date	01/2017–02/2018	01/2017–06/2018	04/2017–03/2018	01/2017–12/2017	01/2017–02/2018
Median days between onset and CSF collection (range)	3 (0–26)	5 (0–33)	1 (0–5)	4.5 (0–61)	3 (0–30)
Antibiotic usage, *n* (%)					
Yes	137 (71)	0	0	12 (40)	56 (35)
No	56 (29)	121 (100)	1	18 (60)	104 (65)
CSF white blood count (cells/mm^3^), *n* (%)					
< 10	179 (90)	139 (10)	23 (74)	8 (27)	270 (89)
10–100	10 (5.0)	0	6 (19)	9 (30)	27 (8.9)
> 100	11 (5.5)	0	2 (6.5)	13 (43)	6 (2.0)
Cerebrospinal fluid glucose (mg/dL), *n* (%)					
< 40	22 (18)	8 (5.8)	6 (40)	11 (37)	24 (9.1)
40–100	101 (80)	126 (91)	9 (60)	19 (63)	191 (72)
> 100	3 (2.4)	5 (3.6)	0	0	50 (19)
Outcome at discharge, *n* (%)					
Died	5 (2.4)	22 (16)	5 (16)	4 (13)	23 (11)
Discharged alive	196 (96)	103 (75)	13 (42)	22 (71)	164 (82)
Transferred	1 (0.5)	0	13 (42)	3 (9.7)	0
Left against medical advice	1 (0.)	12 (8.8)	0	0	14 (7.0)
Pending discharge	2 (1.0)	0	0	2 (6.5)	0

* Although there were a total of 1,535 suspected meningitis cases reported during the study period, 1088 CSF specimens were sent to the WHO RRL. Of these 1088 CSF specimens, 717 had sufficient volume for TAC testing. lumbar puncture was performed.

† This excludes the six samples that failed in the TAC analysis.

‡ Not all the information was available for all the patients. Percentage was calculated for the samples with the corresponding information available.

### Pathogen detection by TAC.

**Figure 1.**
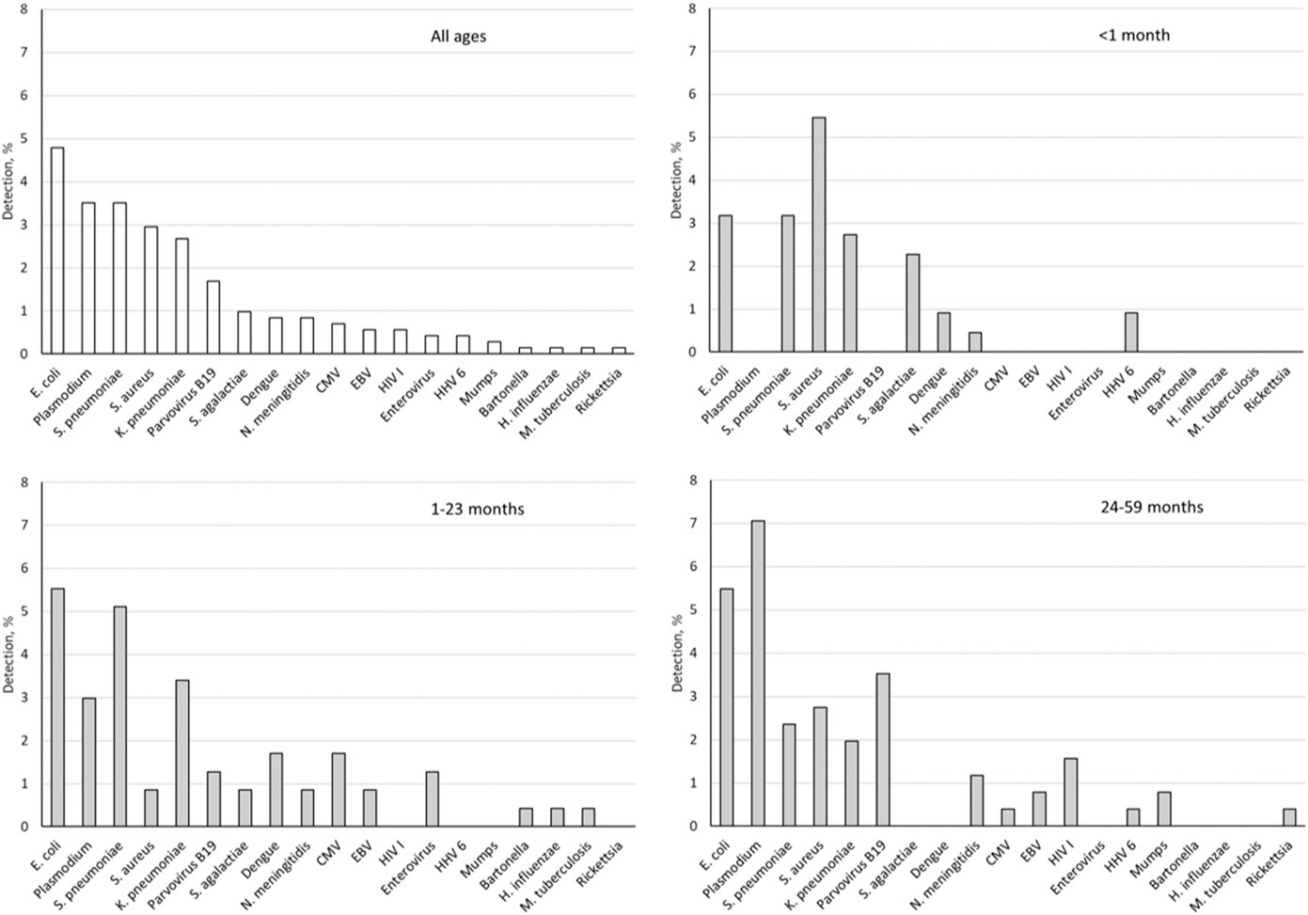
Overall pathogen detection in different age-groups among West African children younger than 5 years with suspected meningitis.

Overall, 20% of the samples had at least one pathogen detected, including 10 bacterial pathogens, eight viral pathogens, and *Plasmodium*. The pathogen detection rate was 22% for Ghana, 24% for Nigeria, 29% for Niger, 45% for Senegal, and 8% for Togo. *Escherichia coli* was the most prevalent (4.8%, Figure 1), followed by *S. pneumoniae* (3.5%) and *Plasmodium* (3.5%). One pathogen was detected in 15.6% of specimens, whereas 4.4% of specimens contained > 1 pathogen. The period from illness onset to CSF collection was 3 days (interquartile range: 1–6). There was no difference in the duration of illness in those with pathogens detected versus those without (*P* > 0.05).

### Comparison of TAC results with the triplex real-time PCR results.

CSF culture was performed on 707 fresh CSF samples, for an overall 2.0% pathogen detection rate. Compared with the culture results, TAC detected the only *H. influenzae* case, all three *S. pneumoniae* cases, and one of the two *N. meningitidis* cases confirmed by culture (see Supplemental Table).

In addition, 711 CSF samples were previously tested for *N. meningitidis*, *H. influenzae*, and *S. pneumoniae* using a triplex real-time PCR assay. The human *RNAse P* gene internal control failed in 249 specimens, and these results were censored according to the protocol. Of the 462 specimens with conclusive results, there were nine positive results by triplex real-time PCR. TAC detected 3/3 *N. meningitidis*, 4/5 *S. pneumoniae*, and 0/1 *H. influenzae* results; however, this latter specimen had a high Cq of 35.8 by triplex real-time qPCR, indicative of a low *H. influenzae* bacterial load. Therefore, TAC yielded an overall sensitivity of 78% (7/9) and 99% specificity (1,358/1,377) versus triplex real-time PCR yet detected an additional 17 *S. pneumoniae* and two *N. meningitidis* cases. TAC also detected an additional 22 *S. pneumoniae* and four *N. meningitidis* cases compared with culture. Thus, the overall yield of a potential etiology was 20% (143/711) by TAC versus 2.0% by culture and 2.4% by triplex real-time PCR.

### Pathogen detection by country and by age.

Table 2 shows the distribution of pathogens in the five countries. Bacteria were the dominant pathogens across all the sites, except Togo, where *Plasmodium* was the leading pathogen detected in 5.0% of the specimens. The most common pathogen was *S. pneumoniae* in Ghana (6.3%) and Niger (13%), whereas it was *E. coli* in Nigeria (14%) and Senegal (16%). Among 220 neonates (aged 0–28 days), 17% had at least one pathogen detected, dominated by the gram-negative bacterial pathogens (Figure 1). *Escherichia coli*, *K. pneumoniae*, and *S. pneumoniae* were consistently detected across all age-groups. *Plasmodium* was exclusively detected in 1- to 59-month-old children. Similarly, more viruses were detected in older age-groups, in particular parvovirus B19.

**Table 2:** Pathogen detection (%) in the five countries

	Ghana (*n* = 205)	Nigeria (*n* = 139)	Niger (*n* = 31)	Senegal (*n* = 31)	Togo (*n* = 305)
*Escherichia coli*	5 (2.4)	**20 (14)**	–	**5 (16)**	4 (1.3)
*Plasmodium*	3 (1.5)	5 (3.6)	2 (6.5)	–	**15 (4.9)**
*Streptococcus pneumoniae*	**13 (6.3)**	4 (2.9)	**4 (13)**	3 (9.7)	1 (0.3)
*Staphylococcus aureus*	8 (3.9)	2 (1.4)	–	3 (9.7)	8 (2.6)
*Klebsiella pneumoniae*	7 (3.4)	8 (5.7)	2 (6.5)	2 (6.5)	–
Parvovirus B19	–	8 (5.7)	1 (3.2)	1 (3.2)	2 (0.7)
*Streptococcus agalactiae*	6 (2.9)	1 (0.7)	–	–	–
Dengue	3 (1.5)	–	–	1 (3.2)	2 (0.7)
*Neisseria meningitidis*	1 (0.5)	1 (0.7)	3 (9.7)	–	1 (0.3)
CMV	2 (1.0)	–	–	1 (3.2)	2 (0.7)
EBV	–	2 (1.4)	1 (3.2)	–	1 (0.3)
HIV I	–	1 (0.7)	1 (3.2)	–	2 (0.7)
Enterovirus	3 (1.5)	–	–	–	–
HHV 6	2 (1.0)	–	–	–	1 (0.3)
Mumps	1 (0.5)	–	–	–	1 (0.3)
HIV II	–	–	–	–	1 (0.3)
*Bartonella*	1 (0.5)	–	–	–	–
*Haemophilus influenzae*	–	–	1 (3.2)	–	–
*Mycobacterium tuberculosis*	–	1 (0.7)	–	–	–
*Rickettsia*	–	1 (0.7)	–	–	–
Total pathogen detection rate	45 (22)	33 (24)	9 (29)	14 (45)	24 (8.0)

Bold values indicate the most prevalent pathogen for each country.

### Effect of antibiotics usage on pathogen detection.

Antibiotic usage information was available for 71% of the patients. Forty-one percent of patients reported antibiotic treatment before admission, and the reported usage varied greatly by site. Although overall bacterial pathogen detection was similar with or without antibiotics, there were considerable differences for *E. coli* and *Plasmodium* detection, with lower rates in those that reported having received antibiotics: 3.4% versus 8.0% for *E. coli* (*P* = 0.038) and 0.5% versus 3.7% for *Plasmodium* (*P* = 0.033).

### Other laboratory testing.

Probable meningitis was pre-classified as a suspected meningitis case with turbid appearance or WBC > 100 cells/mm^3^ or a WBC 10–100 cells/mm^3^ with elevated protein (> 100mg/dl) or decreased glucose (< 40mg/dL).^32^ Among the 20 CSF samples from these probable meningitis cases, a high rate (55%, 11/20) were positive for bacterial pathogens, including one *E. coli*, five *K. pneumoniae*, one *N. meningitidis*, and four *S. pneumoniae*. In addition, the pathogen loads (indicated by the real-time PCR Cq) in these specimens were significantly higher than the quantity in those with low white blood cell counts and high glucose concentration (Mann–Whitney U test, *P* = 0.035). As expected, among samples with low white blood count (< 10 cells/mm^3^), the detection rate was 19% (117/618). We included some *N. meningitidis* serogrouping on the TAC as well. Of the six *N. meningitidis* positives, we identified one serogroup A (Ghana), three serogroup C (one from Togo and two from Nigeria), one serogroup W (Niger), and one untyped likely due to low target concentration (Nigeria).

### Pathogen association with mortality.

Among the 605 patients with outcome at discharge information, 9.8% died, with the lowest rate in Ghana (2.4%) and the highest in Niger and Nigeria (16% each). Table 3 shows the mortality rates by pathogen. Detection of HIV and *Plasmodium* was associated with the highest fatality rates of 50% and 29%, respectively.

**Table 3:** Pathogen association with mortality

	Total	Fatality rate before discharge (%)
CMV	4	25
Dengue	5	0
*Escherichia coli*	28	18
Enterovirus	3	0
HHV 6	3	33
HIV	4	50
*Klebsiella pneumoniae*	15	20
*Neisseria meningitidis*	5	0
Parvovirus B19	10	10
*Plasmodium*	17	29
*Streptococcus agalactiae*	7	14
*Staphylococcus aureus*	19	11
*Streptococcus pneumoniae*	23	17
No pathogen	414	8

## DISCUSSION

Childhood meningitis poses a major public health challenge globally and is particularly problematic because it has diverse infectious etiologies. This work shows the increased diagnostic yield of using a pan-molecular syndromic TAC, which revealed a diversity of 19 pathogens in CSF from five countries in West Africa. Using the TAC assay increased the diagnostic yield of meningitis to 20%, an approximate 10-fold increase over bacterial culture or a triplex real-time PCR assay.^25,26^ The sensitivity of TAC versus the earlier methods that were performed was very good, and many more detections were found with TAC.

Several findings were notable. First, the TAC assay greatly increased the detection of the pneumococcus versus the prior triplex real-time PCR that was performed. Both the triplex real-time PCR assay and the TAC assay targeted the autolysin encoding gene (*lytA*) of the *S. pneumoniae.* Therefore, the improved pneumococcal detection with TAC was likely related to the greater volume of CSF tested and the DNA extraction protocol. The pneumococcus is an extremely important pathogen, was common across the age strata, and the most common pathogen detected in CSF in Ghana and Niger. Ghana had an earlier introduction of the 13-valent PCV (PCV13) in 2012, achieving coverage of 90% by 2016. However, Ghana has experienced large outbreaks of pneumococcal meningitis after PCV introduction.^24^ Implementation of the TAC assay in routine surveillance would enhance the monitoring of pneumococcal meningitis in the post-PCV era across the subregion. To further enhance such surveillance, it would be useful to examine serotypes using direct molecular tools,^33^ and these can be incorporated into a future TAC version. Worth noting is that TAC is easy to use and modular for customized use,^8^ with a reagent cost of ∼$60/sample and 3-hour running time from sample to results.

Over the last century, populations in Africa’s meningitis belt, which includes Senegal, Togo, Niger, and the northern regions of Ghana and Nigeria, have borne the brunt of meningococcal meningitis outbreaks.^34^ Serogroup A meningococcus was the dominant cause of meningitis outbreaks until the rollout of MenAfriVac across countries in the meningitis belt between 2010 and 2018.^5^ However, the emergence of serogroup C, W, and X strains with epidemic potential threaten the success of MenAfriVac.^35–38^ Using the TAC assay, we confirmed one serogroup W and three serogroup C meningitis cases. A meningococcal serogroup A meningitis case was also detected and warrants further investigation. The meningococcal capsular typing included in the TAC assay could be of great value during outbreak emergencies because reactive vaccination campaigns depend on rapid and accurate identification of the meningococcal serogroup causing the outbreak.^39^

The TAC assay also highlighted the potential importance of *E. coli*, particularly in Nigeria and Senegal. *Escherichia coli* is well known to be a common cause of neonatal meningitis.^40^ The high rate of gram-negative pathogens such as *E. coli* and *K. pneumoniae*, particularly in older age categories, was notable. Many of these specimens had highly meningitic CSF indices; thus, we suspect these PCR diagnoses are accurate. Although gram-negative meningitis is less common in older children, comorbidities such as malnutrition may have contributed to this finding.^41,42^ *Escherichia coli* culture–positive rates of 1–16% have been reported in other studies of childhood meningitis in Nigeria and Ghana.^43–45^ The gram-negative bacteria causing meningitis is also concerning because they are associated with the highest rates of antimicrobial resistance.^46^

*Staphylococcus aureus* was also quite common, particularly in the first month of life. This pathogen is often associated with meningitis and sepsis in the neonatal period, particularly in low birth weight infants (information we did not collect).^47^ However, *S. aureus* has been commonly reported as a cause of meningitis by culture in recent reports from Nigeria.^48^

Beyond bacteria, there were a number of additional interesting findings. There was substantial *Plasmodium* in the CSF. We suspect these *Plasmodium* detections reflected cerebral malaria, particularly given the high mortality. Indeed, cerebral malaria has been noted to be a major etiology in children with suspected meningitis,^49^ but it is usually diagnosed based on blood smear and clinical grounds. Notably, *Plasmodium* was not detected in individuals with meningitic CSF profiles, suggesting this was not meningitis per se. Although the detection of malaria in CSF could simply be due to blood contamination of CSF, we think this is unlikely. In one study, the detection of *Plasmodium* HRP-2 protein in CSF, but not plasma, was associated with high mortality in cerebral malaria; thus, DNA detection in the CSF is quite plausible.^50^ Parvovirus B19 was quite common in older children and is well known to be able to cause meningitis.^51^ Group B *Streptococcus* meningitis was also detected and found predominantly among neonates, which is consistent with previous findings.^14,52^

This study has several limitations. First, this study used sentinel-based disease surveillance. Specimens collected through nationwide case-based surveillance systems for meningitis would have been ideal; however, this type of surveillance for meningitis is not operational in most of the West African countries active in the PBM surveillance. The MenAfriNet consortium which has supported the implementation of case-based meningitis surveillance in Burkina Faso, Chad, Mali, Niger, and northern Togo has demonstrated the utility of such efforts.^53^ The representativeness of the findings is also limited in that not all the CSF specimens were available for testing; for instance, only 19% of specimens from Senegal were available for TAC testing. There were also large differences in the age distributions of children presenting with suspected meningitis across the various countries. This means that any intercountry comparisons need to be interpreted with caution. Most of the suspected cases had white blood cell counts less than 10 cells/mm^3^ and glucose > 40 mg/dL, which are not suggestive of probable meningitis. There may be several explanations for this which include overlap of the WHO meningitis case definition with other serious febrile illness. This could certainly be the case for Togo, where the dominant pathogen detected was *Plasmodium*. High rates of antibiotic treatment before lumbar puncture (e.g., 71% in Ghana) could also contribute to low white blood cell counts in children meeting the case definition.^9^

We found that 4.4% of specimens had coinfections, with the most common combinations including *Plasmodium* and a bacterium or a virus (CMV or EBV). Although there is evidence that viral and bacterial coinfections do occur in meningitis patients and may be associated with higher mortality,^54^ these findings warrant thorough investigation in future studies. The significance of CMV also needs further investigation.^55^

In summary, this meningitis TAC provided an abundance of useful information beyond culture and routine triplex PCR assays to understand the etiologies of meningitis in West African children. A cornerstone to defeating meningitis by 2030^7^ will be to ascertain the various causes of meningitis and implement the appropriate control and prevention strategies. Enhanced meningitis case ascertainment could be achieved through the implementation of better molecular detection in meningitis surveillance programs.

## Supplemental files

Supplemental materials

## References

[1] Brandtzaeg P van Deuren M, 2012 Classification and pathogenesis of meningococcal infections. Methods Mol Biol 799: 21–35. 21993637 10.1007/978-1-61779-346-2_2

[2] Collaborators GBDM, 2018 Global, regional, and national burden of meningitis, 1990–2016: a systematic analysis for the Global Burden of Disease Study 2016. Lancet Neurol 17: 1061–1082. 30507391 10.1016/S1474-4422(18)30387-9 PMC6234314

[3] Ramakrishnan M Ulland AJ Steinhardt LC Moisi JC Were F Levine OS, 2009 Sequelae due to bacterial meningitis among African children: a systematic literature review. BMC Med 7: 47. 19751516 10.1186/1741-7015-7-47 PMC2759956

[4] Wahl B 2018 Burden of *Streptococcus pneumoniae* and *Haemophilus influenzae* type b disease in children in the era of conjugate vaccines: global, regional, and national estimates for 2000–15. Lancet Glob Health 6: e744–e757. 29903376 10.1016/S2214-109X(18)30247-X PMC6005122

[5] Trotter CL Lingani C Fernandez K Cooper LV Bita A Tevi-Benissan C Ronveaux O Préziosi MP Stuart JM, 2017 Impact of MenAfriVac in nine countries of the African meningitis belt, 2010–15: an analysis of surveillance data. Lancet Infect Dis 17: 867–872. 28545721 10.1016/S1473-3099(17)30301-8

[6] Mwenda JM 2019 Pediatric bacterial meningitis surveillance in the World Health Organization African region using the invasive bacterial vaccine-preventable disease surveillance network, 2011–2016. Clin Infect Dis 69 (Suppl 2): S49–S57. 31505629 10.1093/cid/ciz472 PMC6736400

[7] WHO, 2018 Defeating Meningitis by 2030: First Meeting of the Technical Taskforce. Available at: https://www.who.int/immunization/research/Defeating_meningitis_2030_TTFJuly2018_report.pdf. Accessed July 1, 2019.

[8] Liu J 2014 Development and assessment of molecular diagnostic tests for 15 enteropathogens causing childhood diarrhoea: a multicentre study. Lancet Infect Dis 14: 716–724. 25022434 10.1016/S1473-3099(14)70808-4

[9] Sacchi CT RT-PCR Surveillance Project Team, 2011 Incorporation of real-time PCR into routine public health surveillance of culture negative bacterial meningitis in Sao Paulo, Brazil. PLoS One 6: e20675. 21731621 10.1371/journal.pone.0020675 PMC3120771

[10] Heinsbroek E Ladhani S Gray S Guiver M Kaczmarski E Borrow R Ramsay M, 2013 Added value of PCR-testing for confirmation of invasive meningococcal disease in England. J Infect 67: 385–390. 23796865 10.1016/j.jinf.2013.06.007

[11] Moore CC Jacob ST Banura P Zhang J Stroup S Boulware DR Michael Scheld W Houpt ER Liu J, 2019 Etiology of sepsis in Uganda using a quantitative polymerase chain reaction-based TaqMan array card. Clin Infect Dis 68: 266–272. 29868873 10.1093/cid/ciy472 PMC6321855

[12] Abade A 2018 Use of TaqMan array cards to screen outbreak specimens for causes of febrile illness in Tanzania. Am J Trop Med Hyg 98: 1640–1642. 29611511 10.4269/ajtmh.18-0071 PMC6086183

[13] Velaphi SC 2019 Surveillance for incidence and etiology of early-onset neonatal sepsis in Soweto, South Africa. PLoS One 14: e0214077. 30970036 10.1371/journal.pone.0214077 PMC6457488

[14] Saha SK 2018 Causes and incidence of community-acquired serious infections among young children in south Asia (ANISA): an observational cohort study. Lancet 392: 145–159. 30025808 10.1016/S0140-6736(18)31127-9 PMC6053599

[15] Liu J 2016 Use of quantitative molecular diagnostic methods to identify causes of diarrhoea in children: a reanalysis of the GEMS case-control study. Lancet 388: 1291–1301. 27673470 10.1016/S0140-6736(16)31529-X PMC5471845

[16] Sonko MA 2019 Changes in the molecular epidemiology of pediatric bacterial meningitis in Senegal after pneumococcal conjugate vaccine introduction. Clin Infect Dis 69 (Suppl 2): S156–S63. 31505635 10.1093/cid/ciz517 PMC6761315

[17] Renner LA 2019 Hospital-based surveillance for pediatric bacterial meningitis in the era of the 13-valent pneumococcal conjugate vaccine in Ghana. Clin Infect Dis 69 (Suppl 2): S89–S96. 31505622 10.1093/cid/ciz464 PMC6736167

[18] Tsolenyanu E 2019 Etiology of pediatric bacterial meningitis pre- and post-PCV13 introduction among children under 5 years old in lomé, Togo. Clin Infect Dis 69 (Suppl 2): S97–S104. 31505623 10.1093/cid/ciz473 PMC6761369

[19] Tagbo BN 2019 Pediatric bacterial meningitis surveillance in Nigeria from 2010 to 2016, prior to and during the phased introduction of the 10-valent pneumococcal conjugate vaccine. Clin Infect Dis 69 (Suppl 2): S81–S8. 31505626 10.1093/cid/ciz474 PMC6736152

[20] Kourna Hama M 2019 Pediatric bacterial meningitis surveillance in Niger: increased importance of *Neisseria meningitidis* serogroup C, and a decrease in *Streptococcus pneumoniae* following 13-valent pneumococcal conjugate vaccine introduction. Clin Infect Dis 69 (Suppl 2): S133–S139. 31505636 10.1093/cid/ciz598 PMC6761310

[21] Marc LaForce F Ravenscroft N Djingarey M Viviani S, 2009 Epidemic meningitis due to group A *Neisseria meningitidis* in the African meningitis belt: a persistent problem with an imminent solution. Vaccine 27 (Suppl 2): B13–B19. 19477559 10.1016/j.vaccine.2009.04.062

[22] World Health Organization, 2018 WHO position paper, meningococcal a conjugate vaccine: updated guidance, February 2015. Vaccine 36: 3421–3422. 28760613 10.1016/j.vaccine.2017.07.063

[23] WHO, 2014 Meningitis Outbreak Response in Sub-Saharan Africa: WHO Guideline. Geneva, Switzerland: World Health Organization. 25674656

[24] Kwambana-Adams BA 2016 An outbreak of pneumococcal meningitis among older children (≥5 years) and adults after the implementation of an infant vaccination programme with the 13-valent pneumococcal conjugate vaccine in Ghana. BMC Infect Dis 16: 575. 27756235 10.1186/s12879-016-1914-3 PMC5070171

[25] Vuong J 2016 Development of real-time PCR methods for the detection of bacterial meningitis pathogens without DNA extraction. PLoS One 11: e0147765. 26829233 10.1371/journal.pone.0147765 PMC4735509

[26] Wang X 2011 Detection of bacterial pathogens in Mongolia meningitis surveillance with a new real-time PCR assay to detect *Haemophilus influenzae*. Int J Med Microbiol 301: 303–309. 21276750 10.1016/j.ijmm.2010.11.004

[27] Collaborators GM, 2017 Global, regional, and national under-5 mortality, adult mortality, age-specific mortality, and life expectancy, 1970–2016: a systematic analysis for the Global Burden of Disease Study 2016. Lancet 390: 1084–1150. 28919115 10.1016/S0140-6736(17)31833-0 PMC5605514

[28] Onyango CO 2017 Evaluation of a TaqMan array card for detection of central nervous system infections. J Clin Microbiol 55: 2035–2044. 28404679 10.1128/JCM.02469-16 PMC5483905

[29] Diaz MH 2013 Optimization of multiple pathogen detection using the TaqMan array card: application for a population-based study of neonatal infection. PLoS One 8: e66183. 23805203 10.1371/journal.pone.0066183 PMC3689704

[30] Liu J 2016 Optimization of quantitative PCR methods for enteropathogen detection. PLoS One 11: e0158199. 27336160 10.1371/journal.pone.0158199 PMC4918952

[31] Liu J 2016 Development of a TaqMan array card for acute-febrile-illness outbreak investigation and surveillance of emerging pathogens, including ebola virus. J Clin Microbiol 54: 49–58. 26491176 10.1128/JCM.02257-15 PMC4702733

[32] Centers for Disease Control and Prevention (CDC), 2009 Pediatric bacterial meningitis surveillance–African region, 2002–2008. MMWR Morb Mortal Wkly Rep 58: 493–497. 19444153

[33] Pholwat S Sakai F Turner P Vidal JE Houpt ER, 2016 Development of a TaqMan array card for pneumococcal serotyping on isolates and nasopharyngeal samples. J Clin Microbiol 54: 1842–1850. 27170020 10.1128/JCM.00613-16 PMC4922116

[34] Greenwood B, 2006 Editorial: 100 years of epidemic meningitis in West Africa–has anything changed? Trop Med Int Health 11: 773–780. 16771997 10.1111/j.1365-3156.2006.01639.x

[35] Kwambana-Adams BA 2018 Meningococcus serogroup C clonal complex ST-10217 outbreak in Zamfara state, northern Nigeria. Sci Rep 8: 14194. 30242204 10.1038/s41598-018-32475-2 PMC6155016

[36] Sidikou F 2016 Emergence of epidemic *Neisseria meningitidis* serogroup C in Niger, 2015: an analysis of national surveillance data. Lancet Infect Diseases 16: 1288–1294. 27567107 10.1016/S1473-3099(16)30253-5 PMC5737706

[37] Retchless AC 2018 Molecular characterization of invasive meningococcal isolates in Burkina Faso as the relative importance of serogroups X and W increases, 2008–2012. BMC Infect Dis 18: 337. 30021533 10.1186/s12879-018-3247-x PMC6052536

[38] Brynildsrud OB Eldholm V Bohlin J Uadiale K Obaro S Caugant DA, 2018 Acquisition of virulence genes by a carrier strain gave rise to the ongoing epidemics of meningococcal disease in West Africa. Proc Natl Acad Sci USA 115: 5510–5515. 29735685 10.1073/pnas.1802298115 PMC6003489

[39] WHO, 2015 Preparedness for outbreaks of meningococcal meningitis due to *Neisseria meningitidis* serogroup C in Africa: recommendations from a WHO expert consultation. Wkly Epidemiol Rec 90: 633–636. 26591025

[40] Furyk JS Swann O Molyneux E, 2011 Systematic review: neonatal meningitis in the developing world. Trop Med Int Health 16: 672–679. 21395927 10.1111/j.1365-3156.2011.02750.x

[41] Roine I Weisstaub G Peltola H; LatAm Bacterial Meningitis Study Group, 2010 Influence of malnutrition on the course of childhood bacterial meningitis. Pediatr Infect Dis J 29: 122–125. 19934786 10.1097/INF.0b013e3181b6e7d3

[42] Mulla MI Moosajee I Rubidge CJ Moosa A, 1984 Nutritional status of children with pyogenic meningitis. J Trop Pediatr 30: 303–306. 6520896 10.1093/tropej/30.6.303

[43] Kuti BP Bello EO Jegede TO Olubosede O, 2015 Epidemiological, clinical and prognostic profile of childhood acute bacterial meningitis in a resource poor setting. J Neurosci Rural Pract 6: 549–557. 26752902 10.4103/0976-3147.165424 PMC4692015

[44] Nwadioha SI Nwokedi EO Onwuezube I Egesie JO Kashibu E, 2013 Bacterial isolates from cerebrospinal fluid of children with suspected acute meningitis in a Nigerian tertiary hospital. Niger Postgrad Med J 20: 9–13. 23661203

[45] Owusu M Nguah SB Boaitey YA Badu-Boateng E Abubakr AR Lartey RA Adu-Sarkodie Y, 2012 Aetiological agents of cerebrospinal meningitis: a retrospective study from a teaching hospital in Ghana. Ann Clin Microbiol Antimicrob 11: 28. 23035960 10.1186/1476-0711-11-28 PMC3473245

[46] Li G 2020 Towards understanding global patterns of antimicrobial use and resistance in neonatal sepsis: insights from the NeoAMR network. Arch Dis Child 105: 26–31. 31446393 10.1136/archdischild-2019-316816 PMC6951234

[47] Medugu N Iregbu K Iroh Tam PY Obaro S, 2018 Aetiology of neonatal sepsis in Nigeria, and relevance of Group b streptococcus: a systematic review. PLoS One 13: e0200350. 30016358 10.1371/journal.pone.0200350 PMC6049915

[48] Airede KI Adeyemi O Ibrahim T, 2008 Neonatal bacterial meningitis and dexamethasone adjunctive usage in Nigeria. Niger J Clin Pract 11: 235–245. 19140361

[49] Page AL 2017 Aetiology and outcomes of suspected infections of the central nervous system in children in Mbarara, Uganda. Sci Rep 7: 2728. 28578421 10.1038/s41598-017-02741-w PMC5457409

[50] Thakur KT Vareta J Carson KA Kampondeni S Potchen MJ Birbeck GL MacCormick I Taylor T Sullivan DJ Seydel KB, 2018 Cerebrospinal fluid *Plasmodium falciparum* histidine-rich protein-2 in pediatric cerebral malaria. Malar J 17: 125. 29566695 10.1186/s12936-018-2272-y PMC5865338

[51] Barah F Vallely PJ Chiswick ML Cleator GM Kerr JR, 2001 Association of human parvovirus B19 infection with acute meningoencephalitis. Lancet 358: 729–730. 11551584 10.1016/S0140-6736(01)05905-0

[52] Okomo U Akpalu ENK Le Doare K Roca A Cousens S Jarde A Sharland M Kampmann B Lawn JE, 2019 Aetiology of invasive bacterial infection and antimicrobial resistance in neonates in sub-Saharan Africa: a systematic review and meta-analysis in line with the STROBE-NI reporting guidelines. Lancet Infect Dis 19: 1219–1234. 31522858 10.1016/S1473-3099(19)30414-1

[53] Patel JC 2019 MenAfriNet: a network supporting case-based meningitis surveillance and vaccine evaluation in the meningitis belt of Africa. J Infect Dis 220 (Suppl 4): S148–S154. 31671453 10.1093/infdis/jiz308 PMC6853281

[54] Kelly MJ 2012 Epstein-barr virus coinfection in cerebrospinal fluid is associated with increased mortality in Malawian adults with bacterial meningitis. J Infect Dis 205: 106–110. 22075766 10.1093/infdis/jir707 PMC3242746

[55] Leber AL 2016 Multicenter evaluation of BioFire FilmArray meningitis/encephalitis panel for detection of bacteria, viruses, and yeast in cerebrospinal fluid specimens. J Clin Microbiol 54: 2251–2261. 27335149 10.1128/JCM.00730-16 PMC5005480

