# Supplementary material for: Etiology of Pediatric Meningitis in West Africa Using Molecular Methods in the Era of Conjugate Vaccines against Pneumococcus, Meningococcus, and Haemophilus influenzae Type b
Source: Am J Trop Med Hyg. 2020 May 26;103(2):696–703. doi: 10.4269/ajtmh.19-0566 (PMC7410464; doi:10.4269/ajtmh.19-0566)
Supplement: Supplementary file 1 [file tpmd190566.SD1.doc]

| **Country** | **Hib** | **PCV** | **MenAfriVac** |
| --- | --- | --- | --- |
| **Ghana** | 2002 | 2012 | 2012 |
| **Niger** | 2008 | 2014 | 2010 |
| **Nigeria** | 2012 | 2014 | 2011 |
| **Senegal** | 2005 | 2013 | 2012 |
| **Togo** | 2008 | 2014 | 2014 |

**Supplemental File 1: Year of introduction of the Hib conjugate vaccine, PCV and MenAfriVac**

**Data sources**

1. [**http://apps.who.int/immunization_monitoring/globalsummary/timeseries/tswucoveragedtp1.html**](http://apps.who.int/immunization_monitoring/globalsummary/timeseries/tswucoveragedtp1.html)
2. [**https://www.path.org/vaccines/**](https://www.path.org/vaccines/)
3. [**https://www.afro.who.int/sites/default/files/2017-06/oms-ivb-rvap-afro-en-20150408_final_sent140317_0.pdf?ua=1**](https://www.afro.who.int/sites/default/files/2017-06/oms-ivb-rvap-afro-en-20150408_final_sent140317_0.pdf?ua=1)
