# Supplementary material for: Etiology of Pediatric Meningitis in West Africa Using Molecular Methods in the Era of Conjugate Vaccines against Pneumococcus, Meningococcus, and Haemophilus influenzae Type b
Source: Am J Trop Med Hyg. 2020 May 26;103(2):696–703. doi: 10.4269/ajtmh.19-0566 (PMC7410464; doi:10.4269/ajtmh.19-0566)
Supplement: Supplementary file 2 [file tpmd190566.SD2.doc]

**Supplemental File 2. Table of qPCR assays validated on the TaqMan Array Card in this study**

| Pathogen | Gene | Forward | Reverse | MGB probe | Reference |
| --- | --- | --- | --- | --- | --- |
| HIV I | LTR | GCCTCAATAAAGCTTGCCTTGA | GGGCGCCACTGCTAGAGA | CCAGAGTCACACAACAGACGGGCACA | ^1^ |
| HIV II | Protease | CACCACACAGAGAGGCGACAGAGGA | TGACCCTCGATATATGCTTGGACTACTGGTC | TGCTGCACCTCAATTCTCTCTTTGG | ^1^ |
|  |  | CACCATGCAGGGARACGACAGAGGA | TGACCCTCRATGTRTGCTGTGACTACTGGTC |  |  |
|  |  | GACCCTACAAGGAGGTGACRGAGGA | TGACCCTCRATACATGCTTTGACTACTGGTC |  |  |
| Parvovirus B19 | VP2 | AAGCCGTGTGCACCCATT | GTACTGGTGGGCGTTTAGTTACG | TAAACACTCCCCACCGTGC | ^2^, modified |
| H. influenzae B | bcs3 | CAAGATACCTTTGGTCGTCTGCTA | TAGGCTCGAAGAATGAGAAGTTTTG | ATGATATGGGTACATCTGTT | ^3^ |
| N. meningitidis A | sacB | AAAATTCAATGGGTATATCACGAAGA | ATATGGTGCAAGCTGGTTTCAATAG | CAAAGTGCCCTTCCTAC | ^4^, modified |
| N. meningitidis B | synD | GCTACCCCATTTCAGATGATTTGT | ACCAGCCGAGGGTTTATTTCTAC | CATAGTTGTTRCCCATCTCT | ^4^, modified |
| N. meningitidis C | synE | CCCTGAGTATGCGAAAAAAATT | TGCTAATCCCGCCTGAATG | TTTCAATGCTAATGAATACCACCG | ^4^, modified |
| N. meningitidis W | synG | TATTTATGGAAGGCATGGTGTATG | TTGCCATTCCAGAAATATCACC | CTGTAATCATTCGCTCCATA | ^4^, modified |
| N. meningitidis X | xcbB | TGTCCCCAACCGTTTATTGG | TGCTGCTATCATAGCCGCC | TGTTTGCCCACATGAATGGC | ^4^, modified |
| N. meningitidis Y | synF | TCCGAGCAGGAAATTTATGAGAATAC | TTGCTAAAATCATTCGCTCCATAT | TATGGTGTACGATATCCCTATC | ^4^, modified |

References

1. Granade TC, Kodani M, Wells SK, Youngpairoj AS, Masciotra S, Curtis KA, Kamili S, Owen SM, 2018. Characterization of real-time microarrays for simultaneous detection of HIV-1, HIV-2, and hepatitis viruses. J Virol Methods 259: 60-65.

2. Ishikawa A, Yoto Y, Tsugawa T, Tsutsumi H, 2014. Quantitation of human parvovirus B19 DNA in erythema infectiosum and aplastic crisis. J Med Virol 86: 2102-6.

3. Marty A, Greiner O, Day PJ, Gunziger S, Muhlemann K, Nadal D, 2004. Detection of Haemophilus influenzae type b by real-time PCR. J Clin Microbiol 42: 3813-5.

4. Wang X, Theodore MJ, Mair R, Trujillo-Lopez E, du Plessis M, Wolter N, Baughman AL, Hatcher C, Vuong J, Lott L, von Gottberg A, Sacchi C, McDonald JM, Messonnier NE, Mayer LW, 2012. Clinical validation of multiplex real-time PCR assays for detection of bacterial meningitis pathogens. J Clin Microbiol 50: 702-8.
