# Supplementary material for: Etiology of Pediatric Meningitis in West Africa Using Molecular Methods in the Era of Conjugate Vaccines against Pneumococcus, Meningococcus, and Haemophilus influenzae Type b
Source: Am J Trop Med Hyg. 2020 May 26;103(2):696–703. doi: 10.4269/ajtmh.19-0566 (PMC7410464; doi:10.4269/ajtmh.19-0566)
Supplement: Supplementary file 3 [file tpmd190566.SD3.doc]

**Supplemental File 3. Comparison of detection methods for *N. meningitidis, S. pneumoniae* and *H. influenzae***

|  | | ***H.influenzae* TAC +** | ***H.influenzae* TAC -** | ***N. meningitidis* TAC +** | ***N. meningitidis* TAC -** | ***S. pneumoniae* TAC +** | ***S. pneumoniae* TAC -** |
| --- | --- | --- | --- | --- | --- | --- | --- |
| Culture | + | 1 | 0 | 1 | 1 | 3 | 0 |
|  | - | 0 | 701 | 4 | 696 | 22 | 677 |
| Triplex PCR | + | 0 | 1 | 3 | 0 | 4 | 1 |
|  | - | 0 | 461 | 2 | 457 | 17 | 440 |
